# Deciphering the Mechanism of YuPingFeng Granules in Treating Pneumonia: A Network Pharmacology and Molecular Docking Study

**DOI:** 10.1155/2022/4161235

**Published:** 2022-10-15

**Authors:** Bing Huang, Jing Luo, Liu-Yuan Liu, Wu-Sheng Deng, Ke Wang, Hua-Song Lu, Jin-Liang Kong

**Affiliations:** Ward of Pulmonary and Critical Care Medicine, Department of Respiratory Medicine, The First Affiliated Hospital of Guangxi Medical University, Nanning 530022, Guangxi, China

## Abstract

**Objective:**

YuPingFeng Granules (YPFGs) is an herbal formula clinically used in China for more than 100 years to treat pneumonia. Nevertheless, the mechanism of YPFG in pneumonia treatment has not been established. This network pharmacology-based strategy has been performed to elucidate active compounds as well as mechanisms of YPFG in pneumonia treatment.

**Methods:**

First, active compounds of YPFG were identified in the traditional Chinese medicine systems pharmacology (TCMSP) database, and then the targets related to the active compounds were obtained from TCMSP and Swiss Target Prediction databases. Next, using DisGeNET, DrugBank, and GeneCards databases, we got therapeutic targets of pneumonia and common targets between pneumonia targets and YPFG. After that, a protein-protein interaction (PPI) network of pneumonia composed of common targets was built to analyze the interactions among these targets, which focused on screening for hub targets by topology. Then, online software and the ClusterProfiler package were utilized for the enrichment analysis of gene ontology (GO) and Kyoto Encyclopedia of Genes and Genomes (KEGG) data. Finally, the visualization software of Autodock was used for molecular docking among the hub target proteins.

**Results:**

10 hub genes were selected by comparing the GO and KEGG functions of pneumonia targets with those of the common targets of YPFG and pneumonia. By using molecular docking technology, a total of 3 active ingredients have been verified as being able to combine closely with 6 hub targets and contribute to their therapeutic effects.

**Conclusion:**

This research explored the multigene pharmacological mechanism of action of YPFG against pneumonia through network pharmacology. The findings present new ideas for studying the mechanism of action of Chinese medicine against pneumonia caused by bacteria.

## 1. Introduction

Pneumonia is an infectious inflammation of the alveoli, lung interstitium, and distal airways. Its etiologic agents include viruses, bacteria, and other pathogens. Lower respiratory tract infections were the third leading cause of total years of life lost (YLLs) in 2016 [1]. According to the season, region, and population characteristics, pneumonia incidences are between 1.5 and 14.0 cases per 1000 person-years [2–4]. With regards to age, the prevalence of community-acquired pneumonia is U-shaped—adults (66 years of age and above) and children (4 years and below) are susceptible to pneumonia [5]. The age-standardized death rate for lower respiratory tract infections is 41.7 per 100 000 population [6]. More seriously, the coronavirus disease 2019 (COVID-19), which began in December 2019 [7], is still spreading worldwide and causing huge property losses and numerous deaths.

Pneumonia including COVID-19 has resulted in hospital services becoming overwhelmed [8]. Currently, the major therapeutic options for pneumonia are antiviral, antibacterial, antifungal, antihistamine, and antipyretic drugs. With the more and more frequent use of antibiotics, the threat of drug resistance is becoming more and more obvious [9]. The solution to this threat is to develop new antimicrobials and alternative treatment options [10]. In recent years, because of its significant therapeutic effects and fewer side effects, traditional Chinese medicine (TCM) has been widely used in preventing and treating infectious diseases and in enhancing human immunity [11, 12]. Especially in this COVID-19 pandemic, TCM has been commonly applied in treating and preventing COVID-19, with good results [13, 14].

YuPingFeng Granules (YPFGs) are a complex and classic traditional Chinese prescription. It is composed of FangFeng1 (FF), BaiZhu2 (BZ), and HuangQi3 (HQ). YPFG is commonly used to treat inflammation-related diseases, including asthma [15], chronic obstructive pulmonary disease [16], and pneumonia [17]. Through metabolomics and network pharmacology approaches, Li et al. [18] revealed that the immune-modulatory effects of YPFG may be mediated via lipid metabolism regulation. Liu et al. [19] identified that YPFG suppresses NLRP3 inflammasome constituents to ameliorate asthma-associated inflammatory responses. Besides, Li Yan et al. [20] exposed that YPFG has a good clinical effect on the prevention of hospital-acquired pneumonia in patients with acute cerebral vascular diseases, and it can improve the immunological function and relieve the lung qi deficiency symptoms. In addition, the results of Sun et al. [21] confirmed that, on the basis of the use of Azithromycin, combined administration with oral YPFG could enhance the overall clinical efficacy in the treatment of mycoplasma pneumonia in children. However, the underlying mechanism for prescribing YPFG for pneumonia treatment is unknown.

**Figure 1:**
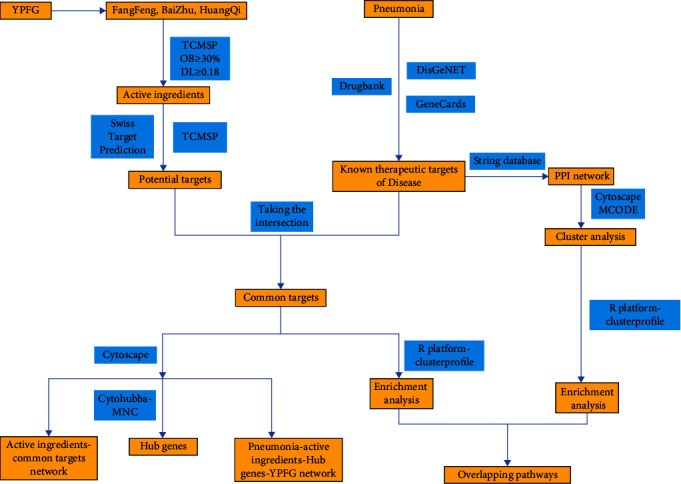
The flowchart of the network pharmacology-based strategy for deciphering the mechanisms of YPFG in pneumonia. The intersections of the active ingredient targets and the disease targets were regarded as common targets. The pivotal active ingredients were obtained through a common targets-active ingredients network analysis. The overlapping pathways were obtained through pathway enrichment analysis of the disease targets and common targets. Abbreviations∶ YPFGs. YuPingFeng Granules; TCMSP: traditional Chinese medicine systems pharmacology; PPI; protein-protein interaction, and OB; oral bioavailability. DL : drug likeness.

To decipher the mechanisms of YPFG on pneumonia, we searched for YPFG targets as well as pathways of YPFG in pneumonia treatment.  Figure 1 shows the research process. The databases used in this study are shown in Supplementary Table 1. In this study, we used a network pharmacology strategy to explore the active compounds of YPFG in the treatment of pneumonia and its regulatory mechanism, and analyzed its important action targets and signal pathways, providing a theoretical basis for the subsequent clinical application of YPFG.

## 2. Materials and Methods

### 2.1. Active Ingredients in YPFG

To screen the active compounds of the 3 herbs in YPFG, a search in TCMSP [22] (updated on May 31, 2014) was carried out by using “FangFeng,” “BaiZhu,” and “HuangQi” as keywords. Normally, traditional Chinese drugs are intragastrically administered. Drug likeness (DL) and oral bioavailability (OB) are affected by drugs absorption through the mucosa of the intestines. Thus, in this study, molecules with a DL index ≥0.18 and OB value ≥33% [23] are considered to be active compounds. Molecular structures, “PubChem CID,” as well as “Canonical SMILES” of active constituents were retrieved from PubChem [24].

### 2.2. Targets Related to Active Ingredients

Targets for active constituents in YPFG were retrieved from the TCMSP database. Considering that the target of the TCMSP database may not be complete, using “Canonical SMILES” of active compounds from PubChem, the targets were searched on Swiss Target Prediction [25], with species being limited to “*Homo sapiens*” and probability set to no less than 0.6. Standardization of identified targets were carried out by searching in UniProt [26].

### 2.3. Known Therapeutic Targets Acting on Pneumonia

Using “pneumonia” as the keyword, we collected pneumonia targets from the DrugBank database (updated on July 2, 2020), GeneCards database (the score of targets were set as no less than 5), and DisGeNET database (the score-gda of targets were set as no less than 0.2). Standardization of these targets were also carried out in UniProt.

### 2.4. Network Construction

#### 2.4.1. Protein-Protein Interaction Data

String [27] is a database of already identified and expected protein-protein interactions (PPIs). Currently, it has about 67.6 million proteins from 14,000 organisms. In this study, target interactions were evaluated via string with “*Homo sapiens*” as the organism and at ≥0.4 as the confidence score. Other parameters were the default settings.

#### 2.4.2. Cluster Analysis

Cluster analyses are aimed at screening similar or same nodes as well as protein complexes from complex PPI networks [28]. Cluster analyses were carried out using the MCODE app in Cytoscape (version 3.9.0) [29] (cutoff = 0.2, maximum depth = 100, *k* core = 2, and degree cutoff = 2).

#### 2.4.3. Network Identification and Visualization of Hub Genes

Considering intersections between pneumonia targets and YPFG, shared targets were identified. A PPI network for disease targets, a PPI network for common targets, and a network for common targets active compounds, as well as a disease-hub gene active compounds network were built and their visualization is carried out by utilizing Cytoscape [30] (version 3.9.0). Hub genes were filtered via cytoHubba, a Cytoscape plug-in. Through the maximum neighborhood component (MNC) approach in cytoHubba, the top 10 genes were generated and considered to be hub genes [31]. The plug-in network analyzer was utilized to conduct interactive network topology assessments. For every node in the interactive network, the degree value is a vital parameter for assessing its topological features, which determines the counts of connections with other nodes, reflecting the significance of a node [32].

### 2.5. GO and KEGG Analyses

GO annotations are utilized to determine the roles of gene products based on three aspects: cell components (CCs), molecular functions (MFs), and biological processes (BPs). KEGG is a database (https://www.kegg.jp/kegg/kegg1.html) integrating chemistry, the genome, and system function data. “ClusterProfiler” in *R* Studio Software was utilized for GO and KEGG analyses, with the screening criterion adjusted to *p* ≤ 0.05[33].

### 2.6. Molecular Docking

According to previous research methods, we conducted molecular docking analysis [34]. We have downloaded the 2D structures of the active compounds from the PubChem database. Moreover, the 2D structures were optimized by Chem3D. The 3D maps of the hub target proteins have been retrieved and downloaded in the PDB database. The target proteins were dewatered, and the original ligands were removed by PyMOL 2.4.0 software. Finally, the visualization software of Autodock is used for molecular docking.

## 3. Results

### 3.1. Potential Targets of Active Compounds

Based on the DL value index and OB value index screening conditions, 45 active constituents were established in YPFG using the TCMSP database. Among them, 18 were obtained from FF, 7 from BZ, and 20 from HQ. Based on the above, active compound targets were obtained by TCMSP and Swiss Target Prediction. After removing duplicate values, we got a total of 252 targets.

### 3.2. Common Targets Active Compounds Network

**Figure 2:**
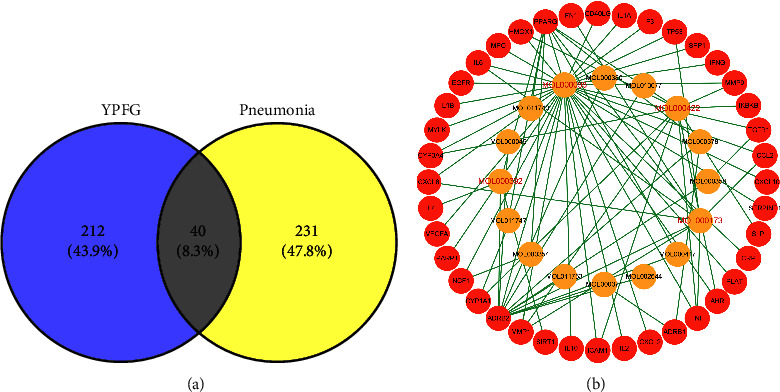
Common targets and common targets-active ingredients network. (a) Common targets of YuPingFeng Granules (YPFG) and pneumonia and (b) common targets-active ingredients network. Red nodes represent the common targets of YPFG and pneumonia and orange nodes represent the active ingredients related to the common targets. The four nodes with red letters in the middle are the compounds with degree ≥4 in the network.

271 pneumonia-associated targets have been identified, by screening databases and removing duplicate targets. By pneumonia-related targets intersecting with active compound targets in Figure 2(a), 40 common targets were obtained.  Figure 2(b) shows the network for active compounds common targets. At ≥4°, we detected four vital active constituents: formononetin (MOL000392), quercetin (MOL000098), kaempferol (MOL000422), and wogonin (MOL000173).

### 3.3. PPI Network and Cluster Analyses of Pneumonia Targets

**Figure 3:**
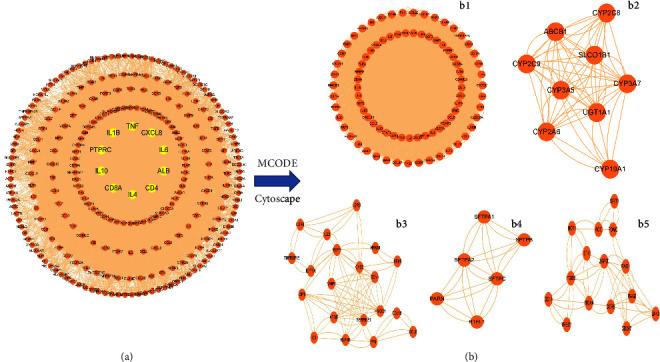
Protein-protein interaction (PPI)network and cluster analysis of the pneumonia targets. (a) PPI network of pneumonia targets. The yellow nodes in the middle represent the top 10 genes with the largest degree values. (b) Top five clustering graphs from the PPI network of pneumonia targets.

**Figure 4:**
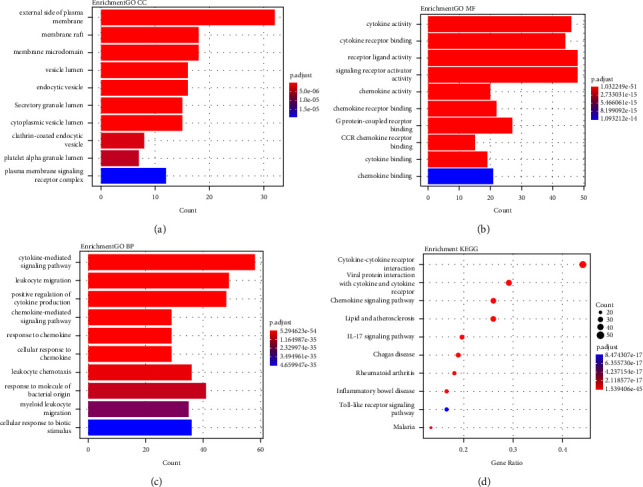
GO and KEGG analysis of pneumonia-related genes. (a) Top 10 significantly enriched terms CC: (b) top10 significantly enriched terms in MF: (c) top 10 significantly enriched terms in BP: and (d) the 10 pathways with the lowest adjusted *p* values. The *X*-axis is the gene ratio of the term, and the *Y*-axis is the name of the terms. The deeper the red color, the smaller the adjusted *p* value. The larger the circle, the greater the number of the target genes in the term.

By using the string database online, we obtained the PPI network pneumonia targets in Figure 3(a). Yellow nodes at the center denote the top 10 genes (that is, TNF, CXCL8, IL-6, ALB, CD4, IL-4, CD8A, IL-10, PTPRC, and IL1B) with the largest degree values, which play vital roles in pneumonia occurrence as well as development. In addition, the PPI network of pneumonia targets were clustered using the MCODE plug-in in Cytoscape, and 9 clusters were found. Based on their scores, we represent the five top clusters in Figure 3(b). GO and KEGG analyses for the targets in the top five clusters were performed. Then, 36 CC, 82 MFs, 1817BPs, and 79 KEGGs pathways were identified (*p* ≤ 0.05).  Figure 4 shows the top 10 significant terms in MFs, BPs, and CCs, as well as the top 10 significant KEGG pathways.

GO enrichment analysis revealed that activation of CCs related to pneumonia occurs on the external side of the plasma membrane, membrane raft, and membrane microdomain. The molecular functions of pneumonia would be highly associated with receptor-ligand activities, cytokine activities, as well as cytokine-receptor binding. The BPs of pneumonia would be highly associated with cytokine-mediated signaling, leukocyte migration, and positive regulation of cytokine production in Figures 4(a)–4(c). The KEGG analyses showed that most pneumonia-associated significant pathways were chemokine signaling pathways, cytokine-cytokine receptor interactions, as well as viral protein interactions with cytokine receptors and cytokines, as shown in Figure 4(d).

### 3.4. Enrichment Analysis of Common Targets

We performed KEGG and GO analyses on the 40 shared targets by the ClusterProfiler software package of the RStudio. Then, 9 CCs, 60 MFs, 1540 BPs, and 73 KEGGs were identified (*p* ≤ 0.05). We obtained top10 significantly enriched terms in BPs and MFs, the top 9 significant terms in CCs, and 10 significantly enriched KEGG pathways (Supplementary Figure 1).

In GO analyses, the targets were highly associated with inflammatory responses and bacteria, including responses to lipopolysaccharide, cellular responses to biotic stimulus, as well as responses to bacterial origin molecules. The CCs were highly enriched in vesicle lumen, platelet granule lumen, and collagen-containing extracellular matrix. The main MFs of the targets were cytokine activity, cytokine-receptor binding, and receptor-ligand activities. The main KEGG pathways of common targets are lipid and atherosclerosis, cytokine-cytokine receptor interactions, fluid shear stress, as well as atherosclerosis.

### 3.5. Identification of Hub Genes

**Figure 5:**
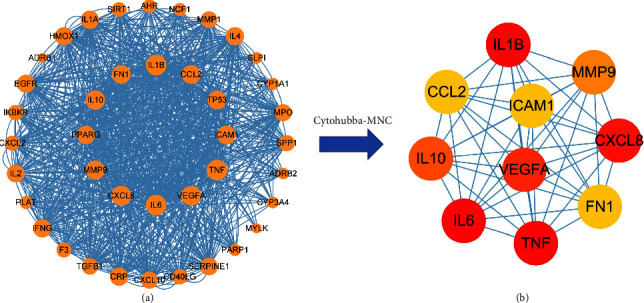
Identification of hub genes of YuPingFeng Granules (YPFGs) for pneumonia. (a) Forty common target protein-protein interaction (PPI) networks. This network has 40 nodes and 932 edges. (b) PPI network of the hub genes, the redder the color, the higher the score and the more it is important.

The PPI network of 40 common targets were obtained from the string database. Through the CytoHubba plug-in in Cytoscape, the top 10 nodes in the network generated by the MNC approach have been considered to be hub genes (Figure 5). The 10 hub genes, namely, ICAM1, MMP9, CCL2, IL1B, TNF, CXCL8, IL10, IL6, FN1, and VEGFA are also important targets of pneumonia.

### 3.6. Analyzing and Selecting the Key GO and KEGG of YPFG on Pneumonia

By comparing the GO and KEGG functions of pneumonia targets with those of the common targets of YPFG and pneumonia, 9 CCs that were overlapping, 30 MFs that were overlapping, 1232 overlapping BPs, and 63 overlapping KEGG pathways were identified. Because hub genes play vital roles in pneumonia treatment via YPFG, we chose overlapping GO, as well as KEGG terms containing hub genes for subsequent analyses. Then, we identified one key cellular component, which contained no less than 3 hub genes, namely, collagen-containing extracellular matrix (GO:0062023). We obtained 17 key biological processes, which contained more than 5 hub genes. We obtained 4 key molecular functions, which contained more than 5 hub genes. We obtained 13 key KEGGs, which contained more than 5 hub genes. The key molecular functions were mainly associated with cytokine activities (GO:0005125), receptor-ligand activity (GO:0048018), and cytokine receptor binding (GO:0005126). The key biological processes were mainly associated with leukocyte migration (GO:0050900), positive regulation of cell adhesion (GO:0045785), and response to lipopolysaccharide (GO:0032496). The key KEGGs were mainly related to Cytokine-cytokine receptor interaction (hsa04060), *Yersinia* infection (hsa05135), IL-17 signaling pathway (hsa04657), and TNF signaling pathway (hsa04668). Although less than five hub genes are enriched in the NF-kappa B signaling pathway (hsa04064) [35], it is important for the occurrence of pneumonia, and we also include it in the analysis next.

### 3.7. Pneumonia-HubGenes-ActiveIngredients-YPFG Network

**Figure 6:**
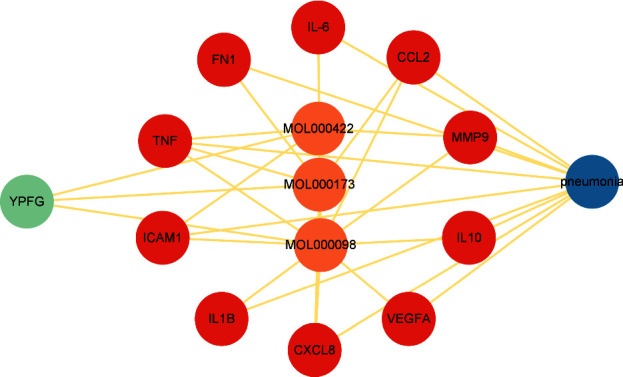
Pneumonia-hubgene-activeingredient-YuPengFeng Granules network. Blue nodes represent pneumonia, green nodes represent YuPengFeng Granules, red nodes represent the hub genes, and orange nodes represent active ingredients.

We build the pneumonia-hubgenes-activecompounds-YPFG network (Figure 6) containing 15 nodes and 30 edges, and contains pneumonia, YPFG, 3 active compounds, and 10 hub genes. Two active compounds, namely, kaempferol (MOL000422) and quercetin (MOL000098) are from HuangQi, and one active compound, namely, wogonin (MOL000173) is from FangFeng.

### 3.8. Molecular Docking

**Figure 7:**
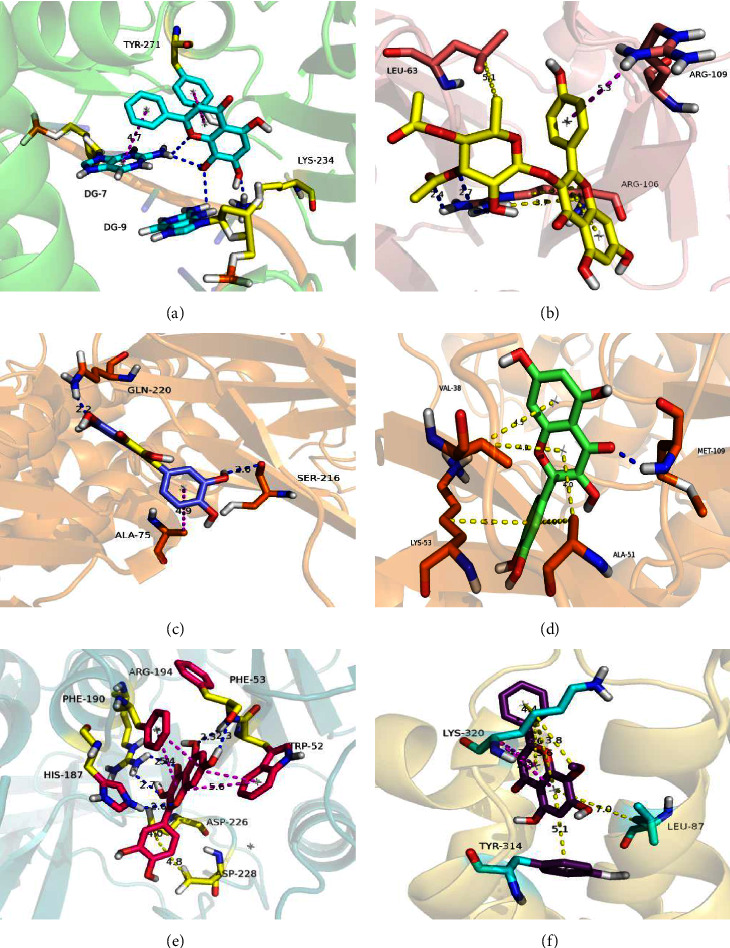
The interaction between active ingredients (ligands) and hub targets (receptors). (a) Wogonin-CCL2, (b) Kaempferol-MMP9, (c) Ouercetin-IL6, (d) Quercetin-IL1B, (e) Quercetin-TNF, and (f) Wogonin-CXCL8. Blue sticks represent hydrogen bonds and yellow sticks indicate hydrophobicity.

Three key active components and some hub genes act as ligands and receptors, respectively. The result of molecular docking is shown in Table 1. As you know, the binding energy between receptor and ligand is less than or equal to -5 kcal/mol, which means the binding is stable [36]. The results displayed that quercetin could stably bind to IL6, IL1B, and TNF. The wogonin stably combined with CCL2 and CXCL8. Besides, kaempferol is bound to MMP9. The interaction between active ingredients (ligands) and hub targets (receptors) is shown in Figure 7.

## 4. Discussion

We investigated the mechanisms of YuPingFeng Granules in pneumonia treatment using network pharmacology. Performing the enrichment analyses of common targets as well as pneumonia targets, respectively, and selecting their overlapping terms, we found that these genes are highly involved in host immune-inflammatory responses, inhibiting cell migration. Li et al. [18] confirmed that YPFG might play an immunomodulatory role by modulating lipid metabolism, thereby affecting bile acid metabolism and inflammatory response. The results of this literature are in agreement with our prediction. Moreover, these genes are associated with the IL-17 signaling pathway (MMP9, CCL2, IL-1B, IL-6, CXCL8, and TNF), TNF signaling pathway (ICAM1, IL-1B, MMP9, IL-6, CCL2, and TNF), Cytokine-cytokine receptor interaction pathway (IL-1B, CCL2, IL-6, CXCL8, TNF, and IL-10) and the *Yersinia* infection pathway (IL-1B, CCL2, FN1, IL-6, CXCL8, IL-10, and TNF), as well as the NF-kappaB signaling pathway (IL-1B, ICAM1, CXCL8, and TNF). By comparing the hub genes with the top ten genes of pneumonia, we found 5 overlapping genes (IL-1B, IL-6, CXCL8, TNF, and IL-10), which might interpret on how the YPFG exerts a therapeutic effect in some types of pneumonia but not all types of pneumonia. Therefore, we will focus on discussing these genes and pathways. In addition, Wang et al. [37] exposed that RELA, TNF, IL-6, MAPK14, and MAPK8 were recognized as key targets of YPFG, which was similar to our analysis.

Pneumonia is associated with infectious inflammations of the lung interstitium, alveoli, as well as distal airways, and its etiologic agents include all kinds of pathogens. Those pathogens induce the host to produce a high abundance of inflammatory factors and cytokines, including IL-6, IL-1B, IL-10, and TNF [38–40]. High IL-6 has been reported in serious diseases, including sepsis [41], acute respiratory distress syndrome (ARDS), and COVID-19 [42]. In healthy individuals, serum IL-6 levels are <4 pg/mL; however, these levels can become elevated in several ways, depending on disease type and severity. In chronic diseases, these levels can reach tens or hundreds of pg/mL, >1000 pg/mL in cytokine storms, while in severe cases, they can reach a level as to be measured in mg/mL [43]. In proinflammatory states, IL-6 binds sIL-6R to form the IL-6–sIL-6R complex that binds glycoprotein 130 (gp130) to amplify inflammatory responses. Moreover, in this process, dysregulated excess and persistent IL-6 synthesis exert pathological effects on acute systemic inflammatory response syndromes and chronic immune-associated diseases, respectively [44].

Lappalainen et al. [45] reported that chronic IL-1B secretion by respiratory epithelial cells of adult mice led to the development of lung inflammation as well as airway fibrosis in adult mice by increasing the production of CXCL1 and MMP-9. A lung gene expression study identified IL1B was significantly up-regulated in COPD small airway epithelial cells and was an airway inflammatory molecule [46]. Through the analysis of the infection mechanism of *Pseudomonas aeruginosa*, Riquelme et al. [47] found substantive IL-1B production and phagocyte pyroptosis induction inhibited bacterial engulfment and destruction, enhancing the proliferation of extracellular bacteria. By means of meta-analysis, Liu et al. [48] found that, relative to nonsevere COVID-19 patients, serum IL‐10, CXCL8, and TNF levels were markedly elevated in severe patients. CXCL8 expression is induced by several cytokines (IL-1, IL-6, and TNF*α*) and bacterial particles and is facilitated by transcriptional factors, activator protein-1 (AP-1), and NF-*κ*B [49]. In addition, through TNF and IL-17 pathways, IL1B could elevate IL-6, IL-17, and TNF levels [50]. Various inflammatory cytokines interactions may amplify the inflammatory response and may produce cytokine storms, and cytokine storms can cause major-organ injury [51]. In general, pneumonia is closely related to the inflammatory response. Controlling excessive inflammatory responses is an important aspect of the treatment of pneumonia.

Through KEGG analyses, it was established that proinflammatory cytokines associated with the IL-17 signaling pathway and synthesis of inflammatory mediators and inflammatory cytokines facilitated by the TNF signaling pathway were associated with inflammatory responses in the lungs. Autoimmune pathology induced by the IL-17 signaling pathway and leukocyte recruitment facilitated by the TNF signaling pathway were associated with host immune responses. Responses to lipopolysaccharides (LPSs) were the highly enriched biological process. As we all know, LPS is a component of the outer wall of the cell wall of Gram-negative bacteria, and the IL-17 signaling pathway mainly occurs in the case of bacterial infection, which suggests that YPFG may be mainly suitable for bacterial pneumonia.

Through constructing the pneumonia-hubgenes-activecompounds-YPFG network, we obtained pivotal active compounds. It is found quercetin (MOL000098) negatively regulates the TLR4 signaling pathway, that is, induced by LPS. Quercetin reduces the expression of TLR4 in peripheral blood mononuclear cells and macrophages of humans and prevents NF-*κ*B from translocating to the nucleus, which improves the anti-inflammatory response [52]. Some researchers believe that Th-1-derived cytokines such as IL-12 improve cellular immunity, while Th2-derived cytokines such as IL-6 have a negative immunomodulatory effect on cellular immunity. Therefore, the beneficial effect of quercetin on immune stimulation is achieved by increasing Th-1-derived cytokines as well as decreasing Th-2-derived cytokines TNF and IL-6 [53]. *In vitro* experiment demonstrates that wogonin (MOL000173) shows a significant neuroprotective effect by inhibiting oxidation and inflammation. These protective effects are through the reduction of caspase-3, TNF, and toll-like receptor 2 expression [54]. *In vivo* experiments in mice suggest that wogonin inhibits the PI3K/Akt/NF-*κ*B signaling pathway, reducing autophagy and inflammation, and then reducing renal tubular cell damage [55].

Kaempferol (MOL000422) downregulates the levels of inflammatory markers, such as TNF*α*, IL-1*β*, and IL-6 by affecting TLR4-dependent NF-*κ*B signaling pathways [56]. A study [57] has demonstrated by western blot and RT qPCR that kaempferol down-regulates the expression of *Staphylococcus aureus* virulence factor Hla. The study also observed that kaempferol shows a good protective effect on MRSA-induced pneumonia in mice. Through the IL-17 signaling pathway, *γδT* cells that can produce IL-17 are shown to control neutrophil influx under infection [58]. Treg cells secrete CXCL8 and promote neutrophil recruitment through CXCL8 [59]. The characteristics of *Yersinia* lung infection are severe pulmonary inflammation and a high abundance of neutrophil infiltration [60] because of the *Yersinia* infection pathway, there is a great risk of death. In our overlapping GO enrichment analysis, leukocyte migration was the most important BP, suggesting that YPFG could inhibit a large number of neutrophils moving to the lung. Molecular docking is a key tool in structural molecular biology and computer-assisted drug design. The goal of ligand-protein docking is to predict the predominant binding mode of a ligand with a protein of a known three-dimensional structure. Successful docking methods search high-dimensional spaces effectively and use a scoring function that correctly ranks candidate dockings. Docking can be used to perform virtual screening on large libraries of compounds, rank the results, and propose structural hypotheses of how the ligands inhibit the target, which is invaluable in lead optimization. Through the verification of molecular docking technology, the 3 active ingredients can combine closely with the hub targets and play a therapeutic role. Meanwhile, the results of molecular docking also exposed the active ingredients of YPFG could tack a part in modulating the progression of pneumonia via MMP9, CCL2, IL-1B, IL-6, CXCL8, and TNF. However, there were still some limitations to the present study. First, due to the slow update of the TCMSP database, the active compounds were not selected enough. Thus, we failed to consider other targets within the datasets. Second, moreover, the lack of validation *in vitro* and *in vivo* experiments is a restriction of the study. Furthermore, investigations are needed to confirm the role of active compounds in pneumonia *in vivo* and to explore its precise mechanisms.

## 5. Conclusion

YPFG may be mainly suitable for bacterial pneumonia. The multitarget action mode of YPFG could effectively treat some types of pneumonia, which are mainly involved in the host immune-inflammatory response and inhibiting cell migration.

For the treatment of pneumonia, traditional Chinese medicine may provide a new direction. Our research has theoretically proved that YPFG can effectively treat pneumonia and provides theoretical support for the continued clinical use of YPFG.

## Figures and Tables

**Table 1:** Docking results between active ingredients and hub genes.

Active ingredients	Hub genes	Binding energy (kcal/mol)
Wogonin	CCL2	−8.2
Kaempferol	MMP9	−5.8
Quercetin	IL6	−7.3
Quercetin	IL1B	−7.3
Quercetin	TNF	−9.4
Wogonin	CXCL8	−7.0

## Data Availability

The original contributions presented in the study are included in the article/Supplementary Material, further inquiries can be directed to the first author or corresponding author.

## Supplementary Materials

Supplementary Materials Supplementary Table 1: using the databases and websites. Supplementary Figure 1: enrichment analysis of common targets.
