# Supplementary material for: Deciphering the Mechanism of YuPingFeng Granules in Treating Pneumonia: A Network Pharmacology and Molecular Docking Study
Source: Evid Based Complement Alternat Med. 2022 Oct 15;2022:4161235. doi: 10.1155/2022/4161235 (PMC9588365; doi:10.1155/2022/4161235)
Supplement: Supplementary Materials — Supplementary Table 1: using the databases and websites. Supplementary Figure 1: enrichment analysis of common targets. [file 4161235.f1.zip › Table 1.docx]

**Supplymentary Table 1** Using the Databases and websites

| Database | website |
| --- | --- |
| Traditional Chinese Medicine Systems Pharmacology database (TCMSP) | https://www.tcmsp-e.com/ |
| PubChem database | https://pubchem.ncbi.nlm.nih.gov/ |
| Swiss Target Prediction database | https://pubchem.ncbi.nlm.nih.gov/ |
| UniProt database | https://www.uniprot.org/ |
| DrugBank database | https://go.drugbank.com/ |
| GeneCards database | https://www.genecards.org/ |
| DisGeNET database | https://www.disgenet.org/ |
| KEGG database | https://www.kegg.jp/kegg/kegg1.html |
| PDB database | https://www.rcsb.org/ |
| String database | https://cn.string-db.org/ |
