# Supplementary figures and images for: Deciphering the Mechanism of YuPingFeng Granules in Treating Pneumonia: A Network Pharmacology and Molecular Docking Study
Source: Evid Based Complement Alternat Med. 2022 Oct 15;2022:4161235. doi: 10.1155/2022/4161235 (PMC9588365; doi:10.1155/2022/4161235)

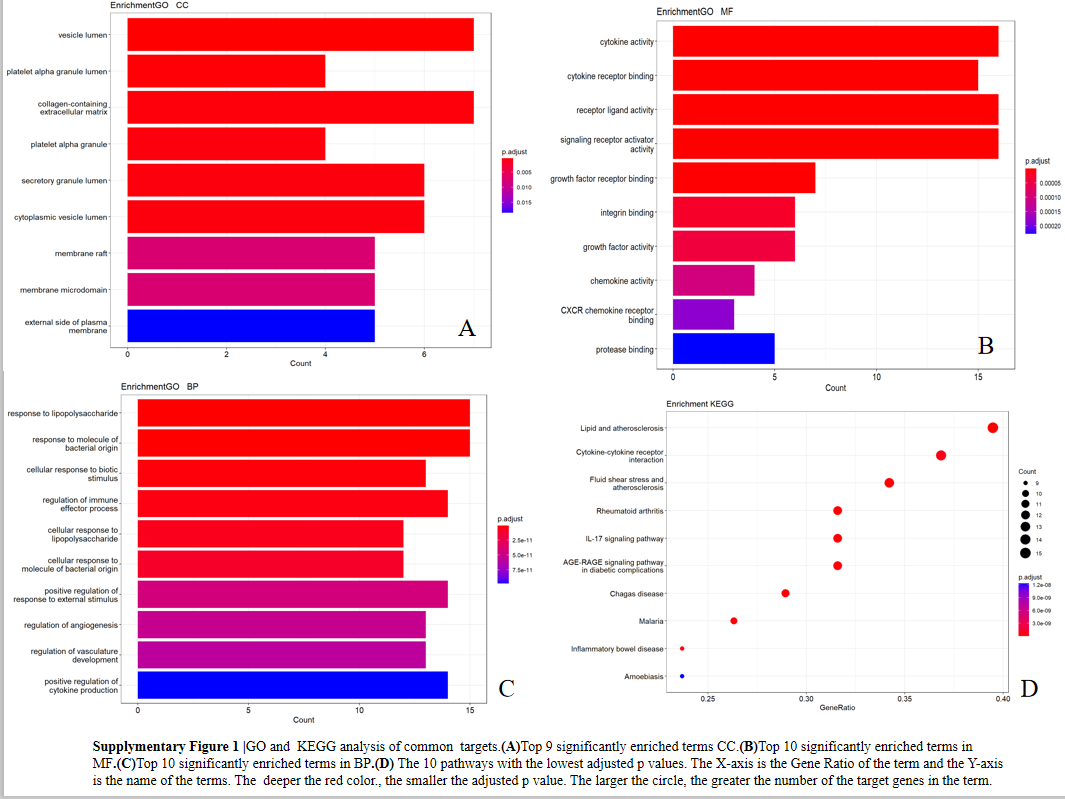

Supplement: Supplementary Materials — Supplementary Table 1: using the databases and websites. Supplementary Figure 1: enrichment analysis of common targets. [file 4161235.f1.zip › Figure 1.png]
